# Supplementary material for: Distilling identifiable and interpretable dynamic models from biological data
Source: PLoS Comput Biol. 2023 Oct 18;19(10):e1011014. doi: 10.1371/journal.pcbi.1011014 (PMC10615316; doi:10.1371/journal.pcbi.1011014)
Supplement: S1 File — (PDF) [file pcbi.1011014.s001.pdf]

Supporting Information for
"Distilling identifiable and interpretable models from biological data"

Gemma Massonis [1], Alejandro F. Villaverde [2,3] and Julio R. Banga [1]
[1]Computational Biology Lab, MBG-CSIC, Pontevedra, Galicia, Spain,
[2]CITMAga, Santiago de Compostela, Galicia, Spain,
[3]Universidade de Vigo, Dept. of Systems and Control Engineering, Vigo, Galicia, Spain
j.r.banga@csic.es, afvillaverde@uvigo.gal

September 13, 2023

1 SINDy-PI: illustration of the method with a case study

In this section, we give a detailed step-wise description of SINDy-PI considering the `Microbial` case study.

As a preamble, before applying the SINDy-PI method to data, it is useful for the sake of clarity (since this is a synthetic problem where we know the ground truth beforehand), to re-arrange the model in an implicit form:

$$\dot{x}_1 = \frac{\mu x_2 x_1}{K_s + x_2} - K_d x_1 = \frac{\mu x_2 x_1 - K_d x_1 (K_s + x_2)}{K_s + x_2} \longrightarrow \dot{x}_1 (K_s + x_2) - (\mu x_2 x_1 - K_d x_1 (K_s + x_2)) = 0 \ ,$$
$$\dot{x}_2 = -\frac{\mu x_2 x_1}{\gamma (K_s + x_2)} \longrightarrow \dot{x}_2 (\gamma (K_s + x_2)) + \mu x_2 x_1 = 0 \ .$$

Reorganizing the above equations we obtain:

$$\dot{x}_1 K_s + \dot{x}_1 x_2 - (\mu + K_d) x_2 x_1 + K_d x_1 K_s = 0 \ ,$$
$$\dot{x}_2 \gamma K_s + \dot{x}_2 x_2 + \mu x_2 x_1 = 0 \ .$$

We now proceed with the first step of SINDy-PI, defining the library of functions. For the sake of compactness and clarity, it suffices to consider a library of terms up to order 3 (higher orders could be considered, achieving the same results, but obfuscating this detailed explanation). For the first state x_1:

$$\Theta_1(x, \dot{x}_1) = [1, x_1, x_2, x_1^2, x_1 * x_2, x_2^2, x_1^3, x_1^2 * x_2, x_1 * x_2^2, x_2^3,$$
$$dx_1, dx_1 * x_1, dx_1 * x_2, dx_1 * x_1^2, dx_1 * x_1 * x_2, dx_1 * x_2^2, dx_1 * x_1^3, dx_1 * x_1^2 * x_2, dx_1 * x_1 * x_2^2, dx_1 * x_2^3]$$

where dx_1 represents $\dot{x}_1$. Similarly, for the second state x_2 we have:

$$\Theta_2(x, \dot{x}_2) = [1, x_1, x_2, x_1^2, x_1 * x_2, x_2^2, x_1^3, x_1^2 * x_2, x_1 * x_2^2, x_2^3,$$
$$dx_2, dx_2 * x_1, dx_2 * x_2, dx_2 * x_1^2, dx_2 * x_1 * x_2, dx_2 * x_2^2, dx_2 * x_1^3, dx_2 * x_1^2 * x_2, dx_2 * x_1 * x_2^2, dx_2 * x_2^3]$$

We can see that using this library and a sparse vector ξ with a few nonzero (nz) elements, we can represent the implicit form of the ground truth model given above:

$$\xi_1 = [0, nz, 0, 0, nz, 0, 0, 0, 0, 0, nz, 0, nz, 0, 0, 0, 0, 0, 0, 0] \ ,$$
$$\xi_2 = [0, 0, 0, 0, nz, 0, 0, 0, 0, 0, nz, 0, nz, 0, 0, 0, 0, 0, 0, 0] \ .$$

The next step in SINDy-PI is to solve the optimization problem using the sequentially thresholded least-squares (STLSQ) algorithm. This method proceeds by iteratively solving the least squares term in the cost function, zeroing out elements of ξ that are below a certain threshold λ. This threshold must be fine-tuned

to select the model that provides the best trade-off between accuracy and efficiency. The procedure we follow involves exploring a finite set of values. In this case, we have considered 19 different values for λ:

$$\lambda \in [10^{-6}, 10^{-5}, 4.0 * 10^{-5}, 0.0002, 0.0001, 0.005, 0.001, 0.05, 0.01, 0.5, 0.1, 1, 1.5, 2, 2.5, 3, 3.5, 4, 4.5]$$

To discover the model, SINDy-PI proceeds by sweeping the library terms for each value of the threshold λ, obtaining a family of possible candidate models. For example, these are the candidate models for the library considered and $\lambda = 10^{-4}$:

LHS term	Candidate model
dx_1	$-(1.0 * (x1 - 7.0 * x1 * x2))/(20.0 * x2 + 20.0)$
$dx_1 * x_1$	$-(1.0 * (x1 - 7.0 * x1 * x2))/(20.0 * x2 + 20.0)$
$dx_1 * x_2$	$-(1.0 * (x1 - 7.0 * x1 * x2))/(20.0 * x2 + 20.0)$
$dx_1 * x_1^2$	$(1.29794e + 7 * x1^3 - 1.03207e + 8 * x1^2 * x2 + 5301860.0 * x1^2 + 2.29226e + 8 * x1 * x2^2 - 1.58715e + 7 * x1 * x2 - 676744.0 * x1 + 5.46752e + 7 * x2^3 - 1.92185e + 7 * x2^2 + 1179750.0 * x2)/(1.6305e + 8 * x1^3 - 3.963e + 9 * x1^2 * x2 - 5.0e + 8 * x1^2 - 1.05801e + 10 * x1 * x2^2 + 3.18384e + 9 * x1 * x2 + 1.6691e + 9 * x2^3 + 3.43262e + 9 * x2^2)$
$dx_1 * x_1 * x_2$	$-(1.0 * (x1 - 7.0 * x1 * x2))/(20.0 * x2 + 20.0)$
$dx_1 * x_2^2$	$-(1.0 * (x1 - 7.0 * x1 * x2))/(20.0 * x2 + 20.0)$
$dx_1 * x_1^3$	$(0.0123782 * x1^3 - 0.262584 * x1^2 * x2 + 0.032249 * x1^2 + 0.31976 * x1 * x2^2 - 0.0147032 * x1 * x2 - 0.00400795 * x1 + 0.0822348 * x2^3 - 0.0392987 * x2^2 + 0.00393562 * x2)/(x1^3 - 7.287 * x1^2 * x2 - 1.71802 * x1^2 - 8.37579 * x1 * x2^2 + 4.61012 * x1 * x2 + 3.76837 * x2^3 + 2.19298 * x2^2)$
$dx_1 * x_1^2 * x_2$	$(3.1291e + 7 * x1^3 - 1.60622e + 8 * x1^2 * x2 + 6.24983e + 8 * x1 * x2^2 - 5.95752e + 7 * x1 * x2 + 1.1326e + 8 * x2^3 - 3.84025e + 7 * x2^2 + 2084930.0 * x2)/(4.4559e + 7 * x1^3 - 1.0e + 10 * x1^2 * x2 - 6.57309e + 8 * x1^2 - 2.69685e + 10 * x1 * x2^2 + 8.72882e + 9 * x1 * x2 + 2.61122e + 9 * x2^3 + 9.29648e + 9 * x2^2)$
$dx_1 * x_1 * x_2^2$	$-(1.0 * (-8651940.0 * x1^3 + 4.8791e + 7 * x1^2 * x2 - 1.51694e + 8 * x1 * x2^2 + 1.19581e + 7 * x1 * x2 - 2.9569e + 7 * x2^3 + 6657890.0 * x2^2))/(1516020.0 * x1^3 - 2.18362e + 9 * x1^2 * x2 - 1.75316e + 8 * x1^2 - 1.0e + 10 * x1 * x2^2 + 2.08931e + 9 * x1 * x2 + 5.49587e + 7 * x2^3 + 3.52251e + 9 * x2^2)$
$dx_1 * x_2^3$	$-(1.0 * (-0.000355306 * x1^3 + 0.0014449 * x1^2 * x2 + 0.00835616 * x1 * x2^2 - 0.00127242 * x1 * x2 + 0.000179851 * x2^3))/(0.000962302 * x1^3 - 0.0908848 * x1^2 * x2 - 0.00791406 * x1^2 + 0.0102132 * x1 * x2^2 + 0.0362678 * x1 * x2 + x2^3 - 0.426411 * x2^2)$

LHS term	Candidate model
dx_2	$-(4.0 * x1 * x2)/(5.0 * x2 + 5.0)$
$dx_2 * x_1$	$-(4.0 * x1 * x2)/(5.0 * x2 + 5.0)$
$dx_2 * x_2$	$-(4.0 * x1 * x2)/(5.0 * x2 + 5.0)$
$dx_2 * x_1^2$	$-(1.0 * (0.00248616 * x1^3 + 0.532359 * x1^2 * x2 - 0.00248627 * x1^2 + 0.904596 * x1 * x2^2 - 0.0963157 * x1 * x2 + 0.000538169 * x1 + 0.0450943 * x2^3 - 0.0201006 * x2^2 + 0.00216938 * x2))/(-0.42376 * x1^3 - 3.76512 * x1^2 * x2 + x1^2 - 7.27708 * x1 * x2^2 + 5.26126 * x1 * x2 + 0.143678 * x2^3 + 3.17677 * x2^2)$
$dx_2 * x_1 * x_2$	$-(4.0 * x1 * x2)/(5.0 * x2 + 5.0)$
$dx_2 * x_2^2$	$-(4.0 * x1 * x2)/(5.0 * x2 + 5.0)$
$dx_2 * x_1^3$	$-(1.0 * (2.86744e + 7 * x1^3 + 1.02292e + 10 * x1^2 * x2 - 2.64499e + 7 * x1^2 + 1.36309e + 10 * x1 * x2^2 - 1.68566e + 9 * x1 * x2 + 4938830.0 * x1 + 6.39996e + 8 * x2^3 - 3.18638e + 8 * x2^2 + 4.11494e + 7 * x2))/(-1.0e + 10 * x1^3 - 6.42074e + 10 * x1^2 * x2 + 2.10531e + 10 * x1^2 - 1.11259e + 11 * x1 * x2^2 + 8.83886e + 10 * x1 * x2 + 1.14426e + 10 * x2^3 + 4.14976e + 10 * x2^2)$
$dx_2 * x_1^2 * x_2$	$-(1.0 * (5891630.0 * x1^3 + 1.08477e + 9 * x1^2 * x2 - 6044420.0 * x1^2 + 2.62916e + 9 * x1 * x2^2 - 2.14497e + 8 * x1 * x2 + 1340880.0 * x1 + 1.87591e + 8 * x2^3 - 6.89413e + 7 * x2^2 + 5084840.0 * x2))/(-6.1136e + 8 * x1^3 - 1.0e + 10 * x1^2 * x2 + 1.7811e + 9 * x1^2 - 2.3976e + 10 * x1 * x2^2 + 1.42852e + 10 * x1 * x2 + 5.64211e + 8 * x2^3 + 1.04053e + 10 * x2^2)$
$dx_2 * x_1 * x_2^2$	$(0.0253272 * x1^2 * x2 + 0.0973572 * x1 * x2^2 - 0.00580322 * x1 * x2 + 0.0109275 * x2^3 - 0.00344962 * x2^2 + 0.00020369 * x2)/(0.0125031 * x1^3 + 0.267131 * x1^2 * x2 - 0.0367751 * x1^2 + x1 * x2^2 - 0.450836 * x1 * x2 + 0.0162831 * x2^3 - 0.467284 * x2^2)$
$dx_2 * x_2^3$	$-(1.0 * (0.00309469 * x1^2 * x2 - 0.0670059 * x1 * x2^2 + 0.00262243 * x1 * x2 - 0.000292367 * x2^3))/(-0.00648915 * x1^3 - 0.091185 * x1^2 * x2 + 0.0134931 * x1^2 + 0.0141565 * x1 * x2^2 + 0.0401987 * x1 * x2 + x2^3 - 0.626075 * x2^2)$

The next step in SINDy-PI is to perform model selection choosing the best trade-off, i.e. the Pareto-optimal model. The total number of candidate models is n_{λ}^{n}, where n_{λ} is the number of thresholds and n the number of state variables. Thus, for this particular example, a total of $19^2 = 361$ candidate models were obtained. The Pareto front is obtained by considering a model complexity metric (such as the Akaike information criterion, AIC), the number of terms, and the score for each candidate model. The following plot shows these metrics for the family of models. The Pareto-optimal model is the one marked with the red rectangle, which has a ξ vector that consists of:

$$\xi_1 = [0, -0.0500, 0, 0, 0.3500, 0, 0, 0, 0, 0, 1, 0, -1.0000, 0, 0, 0, 0, 0, 0, 0] \ ,$$
$$\xi_2 = [0, 0, 0, 0, -0.8000, 0, 0, 0, 0, 0, 1, 0, -1.0000, 0, 0, 0, 0, 0, 0, 0] \ .$$

From this ξ vector and the library, we get the following equations after re-ordering:

$$\dot{x}_1 = 0.35 * x_1 - (0.4 * x_1)/(x_2 + 1.0)$$
$$\dot{x}_2 = -(0.8 * x_1 * x_2)/(1.0 * x_2 + 1.0)$$

This is the final output of SINDy-PI for this case study. Our method then proceeds with the structural identifiability analysis, reparameterization and reformulation steps, as described in the subsection devoted to this problem.

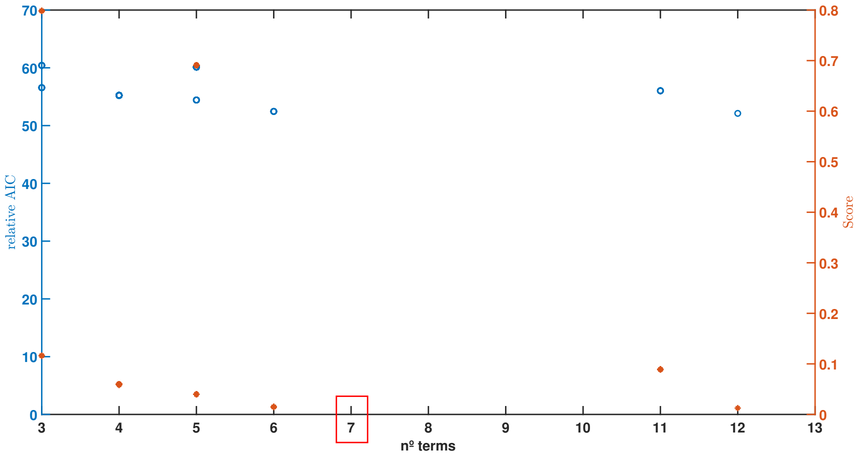

Figure 1: `Microbial case`: Pareto front of the family of models used in model selection.

2 Generation of training data sets

The training data sets for the case studies were generated following the guidelines in [1, 2]. A set of N_{IC} different initial conditions were randomly generated inside a range defined by lower and upper bounds (lb, ub). Data were generated by simulating each model with its nominal parameters and different initial conditions for a certain time horizon (t_0, t_{f}), with sampling (measurement) times given by a time step dt. Next, 10% of the total generated data was selected via random permutation as training data. Finally, a different set of initial conditions (ICS_{pp}) were subsequently used to evaluate the predictive power of the discovered model. Below, we report the values of these settings for each case study.

2.1 `Lorenz`

parameter	a	b	c
value	10	28	8/3

Table 1: `Lorenz`: nominal values of the parameters.

Setting	value
N_{IC}	80
lb	1e-4
ub	0.4706
t_0	0
t_{f}	15
dt	0.1
ICS_{pp}	[0.35,0.2,0.4]

Table 2: `Lorenz`: settings used to generate the training data set and evaluate the predictive power of the discovered model.

2.2 `Immunity`

parameter	a	k	e	β	γ	α	S	d	δ
value	0.18	60	0.005	0.013	0.02	0.01	1.1	0.001	0.105

Table 3: `Immunity`: nominal values of the parameters.

Setting	value
N_{IC}	80
lb	0.0500
ub	75.9614
t_0	0
t_{f}	15
dt	0.1
ICS_{pp}	[0.2635;0.8278]

Table 4: `Immunity`: settings used to generate the training data set and evaluate the predictive power of the discovered model.

2.3 `Bacterial`

parameter	a_1	a_2	a_3	b_1	b_2
value	0.004	0.07	0.04	0.82	1854.5

Table 5: `Bacterial`: nominal values of the parameters.

Setting	value
N_{IC}	80
lb	1e-4
ub	4.9
t_0	0
t_{f}	15
dt	0.1
ICS_{pp}	[0.8;0.9]

Table 6: `Bacterial`: settings used to generate the training data set and evaluate the predictive power of the discovered model.

2.4 `Microbial`

parameter	K_d	K_s	μ	γ
value	0.05	1	0.4	0.5

Table 7: `Microbial`: nominal values of the parameters.

Setting	value
N_{IC}	80
lb	1e-4
ub	0.4706
t_0	0
t_f	15
dt	0.1
ICS_{pp}	[0.8;0.9]

Table 8: `Microbial`: settings used to generate the training data set and evaluate the predictive power of the discovered model.

2.5 `Crypt`

parameter	a_1	a_2	a_3	b_1	b_2	b_3	g	k_0	k_1	m_0	m_1
value	0.1	0.3	0.69	0.1	0.3	0.397	0.139	0.1	3e-4	0.1	4e-4

Table 9: `Crypt`: nominal values of the parameters.

Setting	value
N_{IC}	80
lb	1e-4
ub	79.9582
t_0	0
t_f	15
dt	0.1
ICS_{pp}	[0.35;0.2;0.1]

Table 10: `Crypt`: settings used to generate the training data set and evaluate the predictive power of the discovered model.

2.6 `Glycolysis`

parameter	c_1	c_2	c_3	d_1	d_2	d_3	d_4	e_1	e_2	e_3	e_4	f_1	f_2
value	2.5	-100	13.6769	200	13.6769	-6	-6	6	-64	6	16	64	-13

parameter	f_3	f_4	f_5	g_1	g_2	h_1	h_2	h_3	h_4	h_5	j_1	j_2	j_3
value	13	-16	-100	1.3	3.1	-200	13.6769	128	-32	-32/25	6	-18	-100

Table 11: `Glycolysis`: nominal values of the parameters.

Setting	value
N_{IC}	450
lb	1e-7
ub	0.25
t_0	0
t_{f}	15
dt	0.1
ICS_{pp}	[0.5;0.2;1.1;0.8;0.5;0.4;0.1]

Table 12: `Glycolysis`: settings used to generate the training data set and evaluate the predictive power of the discovered model.

3 Code availability

The code to reproduce the results of this study is available at `https://doi.org/10.5281/zenodo.7713047`. For each case study, we provide a Matlab interactive notebook (live script) which executes the different steps of our workflow. To facilitate reproducibility, we also provide detailed reports (in HTML format) with the results obtained for each case study.

Requirements:

- MATLAB (tested with version R2020b under Win10), including:
  - Symbolic Math Toolbox.
  - Parallel Computing Toolbox (optional).
- STRIKE-GOLDD (tested with version 4.0.2), available at `https://github.com/afvillaverde/strike-goldd`)
- SINDy-PI, available at `https://github.com/dynamicslab/SINDy-PI.git`.

Installation: please follow the instructions in the `README_install.txt` file.

References

[1] Mangan NM, Brunton SL, Proctor JL, Kutz JN. Inferring biological networks by sparse identification of nonlinear dynamics. IEEE Transactions on Molecular, Biological and Multi-Scale Communications. 2016;2(1):52–63.

[2] Kaheman K, Kutz JN, Brunton SL. SINDy-PI: a robust algorithm for parallel implicit sparse identification of nonlinear dynamics. Proc Math Phys Eng Sci. 2020;476(2242):20200279.
